# The safety and efficacy of neoadjuvant immunochemotherapy in locally advanced esophageal squamous cell carcinoma: a meta-analysis and systematic review

**DOI:** 10.3389/fimmu.2026.1687326

**Published:** 2026-02-10

**Authors:** Qing Shen, Haiyang Liu, Huimin Xue, Ailifeiya Yilixiati, Senmiao Yan, Wenya Li, Na Song

**Affiliations:** 1 Department of Medical Oncology, the First Hospital of China Medical University, Shenyang, China; 2 Department of Medical Oncology, the Emergency General Hospital, Beijing, China; 3 Department of Thoracic Surgery, the First Hospital of China Medical University, Shenyang, China; 4 Key Laboratory of Anticancer Drugs and Biotherapy of Liaoning Province, the First Hospital of China Medical University, Shenyang, China

**Keywords:** esophageal squamous cell carcinoma, immunotherapy, meta-analysis, neoadjuvant chemoradiotherapy, neoadjuvant immunochemotherapy

## Abstract

**Purpose:**

The standard treatment for locally advanced esophageal squamous cell carcinoma (ESCC) is neoadjuvant chemoradiotherapy plus esophagectomy; however, there remains a significant risk of distant metastasis following surgery, which compromises long-term survival among patients. The present study involved a meta-analysis designed to explore the safety and efficacy of neoadjuvant immunochemotherapy (nICT) in patients with locally advanced ESCC.

**Methods:**

PubMed, Embase, The Cochrane Library, and Web of Science were searched from inception until June 30, 2025. The extracted data included: pathological complete response (pCR), major pathological response (MPR), objective response rate (ORR), *R*_0_ resection rate, the incidence of adverse events (AEs), and ≥ Grade 3 AEs.

**Results:**

Some 30 studies with a total of 1185 patients were included, wherein the treatment regimen was nICT, without restrictions on the type of immune agents. The results showed that the MPR rate after nICT was 53% (95% confidence interval (CI): 46-59%), and the pooled pCR rate was 32% (95% CI: 29-35%). The pooled *R*_0_ resection was 97% (95% CI: 96-98%), and the pooled ORR rate was 68% (95% CI: 64-72%). The incidence of ≥ Grade 3 treatment-related adverse events (TrAEs) was 26% (95% CI: 17-38%), the incidence of ≥ Grade 3 surgery-related adverse events (SrAEs) was 3% (95% CI: 1-5%), and the main TrAEs in hematological toxicity were leukopenia, neutropenia, and thrombocytopenia. The main symptoms of non-hematological-toxicity TrAEs were nausea, vomiting, fatigue, decreased appetite, and rash. Infection and anastomotic fistula were the most common postoperative complications. In all, 9 cases of surgery-related deaths were identified. Among them, 3 cases were pulmonary complications (all related to pneumonia), 3 cases were direct surgery-related complications (hemorrhagic shock, anastomotic leakage complicated with hemorrhage, and esophagotracheal fistula, respectively), 1 case was severe infection, and 2 cases were attributed to unspecified causes (fatal due to surgery-related Grade V adverse events). Fatal surgical complications were uncommon.

**Conclusions:**

This study preliminarily indicates the efficacy and safety of nICT in locally advanced ESCC in China. This combination regimen exhibits superior pCR with tolerable safety profiles, suggesting a new therapeutic strategy for patients with locally advanced ESCC. (CRD42024574607).

**Systematic review registration:**

https://www.crd.york.ac.uk/PROSPERO/view/CRD42024574607, identifier CRD42024574607.

## 1 Introduction

As one of the most common gastrointestinal malignancies in the world, esophageal cancer (EC) is the sixth leading cause of cancer-related death, mainly concentrated in developing countries and some countries and regions with poor levels of economic development (1). In contrast to Western nations where adenocarcinoma is the predominant pathological type, esophageal squamous cell carcinoma (ESCC), accounting for around 90% of cases, is the predominant subtype of EC in China (2). EC has become a serious threat to the health of Chinese residents because of its hidden onset, and most patients are diagnosed in the middle and advanced stages.

For locally advanced EC, combined surgery after neoadjuvant chemoradiotherapy (nCRT) is the standard treatment strategy. The clinical research results of the CROSS study (3) (enrolled ESCC, adenocarcinoma and undifferentiated carcinoma) and NEOCRTEC 5010 (4) (enrolled 100% ESCC) showed that the *R*_0_ resection in the nCRT group was much higher than in the operation group, and the adverse events (AEs) were generally controllable and similar in each group. In view of the above two large phase III studies, nCRT has become the standard treatment model for locally advanced ESCC and been written into major clinical guidelines. However, the risk of postoperative recurrence and distant metastasis remained high in patients who achieved pathological complete response (pCR) after long-term follow-up (4, 5). Further analysis revealed that nCRT could improve local control of the lesion, but did not reduce the rate of distant metastasis. In addition, a number of studies confirmed that the overall survival (OS) was not prolonged after receiving nCRT. JCOG1109 (6) implied the increase in pCR did not convert into long-term survival benefits for patients. According to the 5-year follow-up data, the neoadjuvant DCF (docetaxel + cisplatin + 5-fluorouracil) group exhibited a statistically significant OS benefit (7). Another study conducted in China comparing nCRT with neoadjuvant chemotherapy (nCT) in locally advanced ESCC also suggested that although the pCR rate was increased in the nCRT group (35.7% *v*. 3.8%, *P* < 0.001), there was no significant difference in the 1-year OS rate between the two groups (8). Moreover, patients in Eastern countries exhibit a poorer pathological response to nCRT compared with Western countries (9). Therefore, it is necessary to explore neoadjuvant therapy models with better safety and clinical operability.

Immunotherapy has led to significant improvements in metastatic settings and postoperative adjuvant therapy in ESCC. Studies of immunotherapy (including programmed cell death protein 1 (PD-1) blockade and programmed cell death ligand 1 (PD-L1) blockade) combined with chemotherapy in the first-line treatment of advanced EC have obtained a higher objective response rate (ORR) ranging from 45%-72.1% (10, 11). The high tumor shrinkage rate brings the possibility of conversion therapy for some advanced patients, therefore more researchers have focused on the application of neoadjuvant immunotherapy in resectable ESCC. In fact, the effectiveness of neoadjuvant immunotherapy has been reported in lung cancer (12). Based on the existing data, this meta-analysis aims to explore the safety and efficacy of neoadjuvant immunochemotherapy (nICT), and provide a basis for future options involving neoadjuvant regimens.

## 2 Materials and methods

### 2.1 Search strategy

Meta-analysis was conducted in accordance with the Preferred Reporting Items for SystematicReviews and Meta-Analyses (PRISMA) guidelines. A thorough search was conducted in PubMed, Web ofScience, Embase, and the Cochrane Library. We also searched the updated unpublished data of ongoing clinical trials of neoadjuvant immunotherapy or chemoimmunotherapy in ESCC from international congresses such as American Society of Clinical Oncology (ASCO), American Association for Cancer Research (AACR), European Society for Medical Oncology (ESMO), and other congresses. The search items included: “Immunotherapy”, “Immunotherapies”, “Neoadjuvant Therapy”, “Neoadjuvant Therapies”, “Therapy, Neoadjuvant”, “Neoadjuvant Treatment”, “Neoadjuvant Treatments”, “Treatment, Neoadjuvant”, “Neoadjuvant Chemoradiotherapy”, “Neoadjuvant Chemoradiation Therapy”, “Treatment, Neoadjuvant Chemoradiation”, “Neoadjuvant Radiation Treatments”, “Neoadjuvant Chemoradiation Treatment”, “Radiation Treatment, Neoadjuvant”, “Esophageal Neoplasm”, “Esophagus Neoplasm”, “Cancer of Esophagus”, “Esophagus Cancer”, “Esophageal Cancers”, “Cancers, Esophageal”, “Treatment, Neoadjuvant Systemic” and so on (**Supplementary Table S1**). The search timeline was from inception to June 30, 2025. The publication search was limited to English language.

### 2.2 Inclusion and exclusion criteria

The inclusion criteria were as follows: (1) pathological evidence confirming the diagnosis of locally advanced ESCC; (2) ESCC patients receiving immunotherapy combined with chemotherapy as neoadjuvant therapy, including but not limited to PD-1/PD-L1, cytotoxic T-lymphocyte-associated protein 4 (CTLA-4), or their combined inhibitors; (3) studies reporting ORR or pCR or major pathological response rate (MPR) or *R*_0_ resection rate; (4) the research type was a prospective experiment; and (5) articles published in English. The exclusion criteria were as follows: (1) reviews, case reports and letters; (2) studies that did not provide results of outcome indexes; (3) animal studies; and (4) the experimental design included only esophageal adenocarcinoma.

### 2.3 Data extraction and quality assessment

Two investigators (Q.S. and H.L.) performed independent data extraction from eligible studies. Disagreements were resolved through discussions between the two investigators. In total, we extracted the following information: name of the first author, publication year, country, sample size, sex, age, study design, treatment, ORR, *R*_0_, pCR, MPR, and AEs. Because the included studies were almost all single-arm trials, the Joanna Briggs Institute (JBI) quality assessment tool was used to assess the quality of the included studies. The main assessment contents included whether there were clear criteria for inclusion of cases, whether standard and credible methods were adopted for diagnosis, whether the included subjects were comprehensive and coherent, and whether the demographic information, clinical information, outcome and follow-up results of the subjects were clearly reported.

### 2.4 Statistical analysis

To assess the safety and effectiveness of nICT in ESCC patients, we calculated the pooled ORR, *R*_0_, pCR and MPR. Heterogeneity among studies was evaluated using the Cochrane Q test and I^2^ test. If the heterogeneity was significant (I^2^ > 50% or *P* < 0.05), the random effect model was adopted; otherwise, the common effect model was used. Sensitivity analysis was performed to evaluate the robustness of this meta-analysis. Publication bias was assessed by Egger’s test, and their symmetry was evaluated via funnel plots. All the statistical analyses were performed using Stata 17.0 and R 4.4.2. A value of *P* < 0.05 was considered statistically significant.

## 3 Results

### 3.1 Literature search

**Figure 1:**
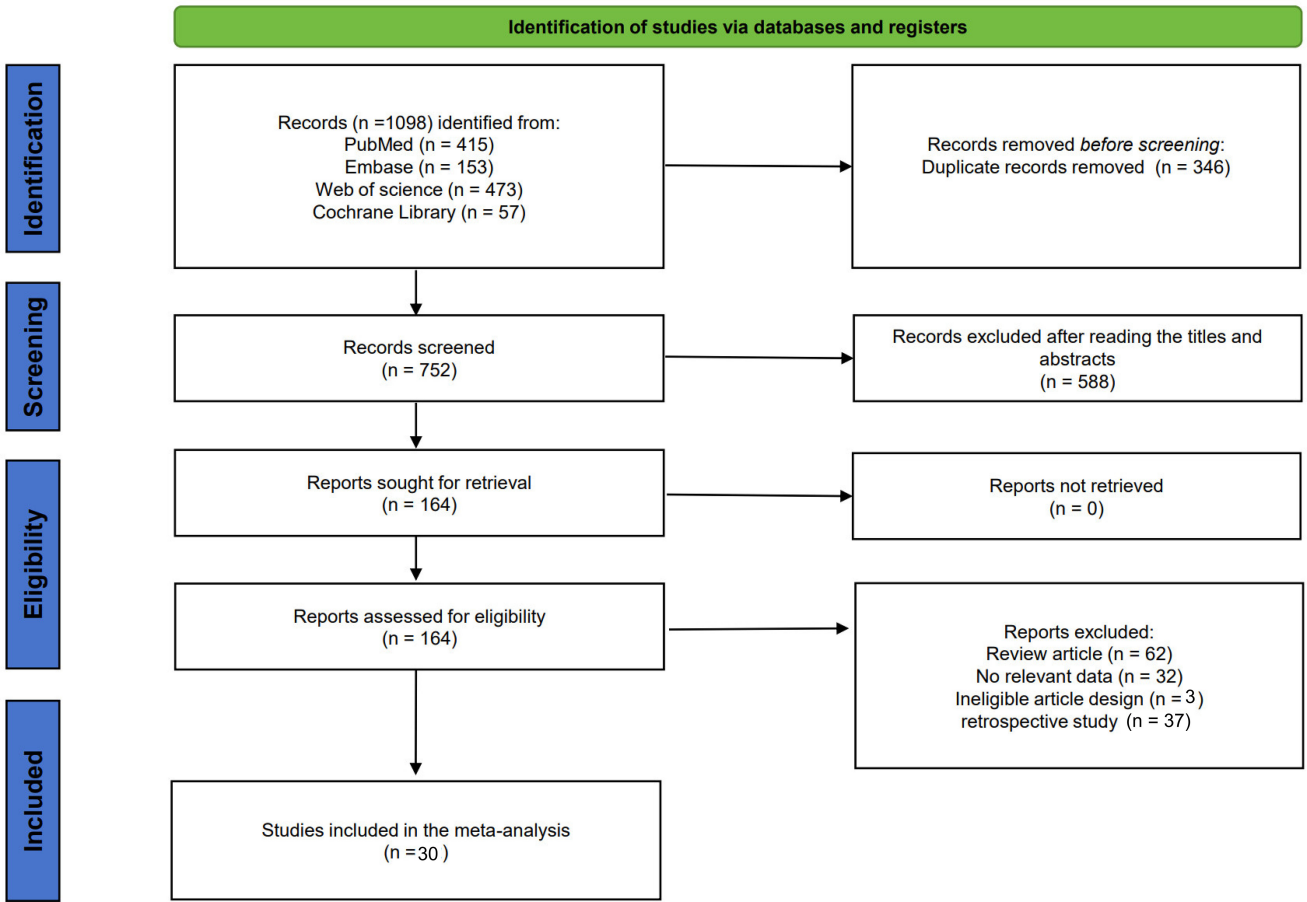
Flowchart of literature search and study selection.

The initial literature search identified 1098 records, and after the removal of duplicate studies, 752 records remained. A total of 588 records were discarded after examining the titles and abstracts, and 164 studies were subsequently evaluated by reading the full text. 134 studies were excluded for the following reasons: review article (n = 62), no relevant data (n = 32), ineligible article (n = 3), and retrospective study (n = 37). Finally, 30 studies with 1185 patients were included in the present meta-analysis (**Figure 1**).

### 3.2 Characteristics of included studies

As shown in **Table 1**, eligible studies had the following baseline characteristics: all included studies were conducted in the Chinese population, with a pathological type of 100% ESCC and no esophageal adenocarcinoma or other subtypes; the results of each study were published in English, and the sample size ranged from 10 to 127; each study used anti-PD-1/PD-L1 antibodies to explore the efficacy and safety of nICT. Investigational immune-checkpoint inhibitors included camrelizumab, pembrolizumab, sintilimab, tislelizumab, durvalumab, socazolimab, adebrelimab, toripalimab, and nivolumab. The risk of bias was assessed using the JBI quality assessment tool (**Table 2**). The main assessment included whether there were clear criteria for inclusion of cases, whether standard and credible methods were adopted for diagnosis, whether the included subjects were comprehensive and coherent, and whether the demographic information, clinical information, outcome, and follow-up results of the subjects were clearly reported.

**Table 1:** Basic characteristics of included studies in this meta-analysis.

Authors	Published year	Study period	Sample size	Country	Study design	Age(years)mean ± SD	Gender(male proportion)	Clinical stages			Treatment
								II	III	IV	
D.Shen (13)	2021	2019-2020	28	China	Single-arm	62.52 ± 7.70	0.964(27/28)	0.143(4/28)	0.75(21/28)	0.107(3/28)	nivolumab/pembrolizumab/camrelizumab
Z.Wu (14)	2021	2019-2020	38	China	Single-arm	62.03 ± 4.21	0.947(36/38)	cT3–4a,N1–3 and M0			pembrolizumab(55.26%)/camrelizumab(31.58%)/sintilimab(13.16%)
P.Yang (15)	2021	2019-2020	16	China	Single-arm	60.9 ± 7.80	0.875(14/16)	0.25(4/16)	0.625(10/16)	0.125(2/16)	camrelizumab
L.Zhao (16)	2021	2019-2020	30	China	Double-arm	−	−	cT3–4 or N+			toripalimab
H.Duan (17)	2022	2019-2020	18	China	Single-arm	61.65 ± 11.80	0.778(14/18)	0.389(7/18)	0.556(10/18)	0.055(1/18)	pembrolizumab
L.Gao (18)	2022	2019-2020	20	China	Single-arm	58.3 ± 5	0.85(17/20)	≥ cT3 or ≥ N+			toripalimab
W.He (19)	2022	2020	20	China	Single-arm	61.4 ± 6.50	0.75(15/20)	−	0.80(16/20)	0.20(4/20)	toripalimab
B.Jiang (20)	2022	2020-2021	10	China	Single-arm	−	−	−			camrelizumab/sintilimab/tislelizumab
J.Liu (21)	2022	2020	56	China	Single-arm	60.01 ± 6.56	0.75(42/56)	0.232(13/56)	0.679(38/56)	0.089(5/56)	camrelizumab
J.Liu (22)	2022	2019-2020	60	China	Single-arm	64.37 ± 5.62	0.833(50/60)		0.85(51/60)	0.15(9/60)	camrelizumab
Z.Wang (23)	2022	−	30	China	Single-arm	59.15 ± 9.80	−	−			camrelizumab
W.Yang (24)	2022	2020	23	China	Single-arm	58.6 ± 10.10	0.957(22/23)	0.348(8/23)	0.652(15/23)	−	camrelizumab
Z.Zhang (25)	2022	2019-2021	47	China	Single-arm	66.71 ± 4.59	0.766(36/47)	0.191(9/47)	0.702(33/47)	0.106(5/47)	sintilimab
X.Chen (26)	2023	2019-2022	30	China	Single-arm	62.10 ± 6.86	0.733(22/30)	0.833(25/30)	0.1(3/30)	0.067(2/30)	sintilimab
J.He (27)	2023	−	26	China	Single-arm	−	−	−			durvalumab
Y.Li (28)	2023	2021	32	China	Double-arm	61.77 ± 5.78	0.719(23/32)	0.375(12/32)	0.594(19/32)	0.031(1/32)	socazolimab
J.Wang (29)	2024	2021-2022	35	China	Single-arm	−	−	−			camrelizumab
G.Yang (30)	2023	2020-2021	47	China	Single-arm	58.8 ± 7.1	0.809(38/47)	0.234(11/47)	0.766(36/47)	−	camrelizumab
Y.Yang (31)	2024	2019-2020	60	China	Single-arm	64.37 ± 5.62	−		0.85(51/60)	0.15(9/60)	camrelizumab
Z.Zhang (32)	2021	2020-2021	30	China	Single-arm	58.3 ± 7.1	0.87(26/30)		0.90(27/30)		sintilimab
X.Yan (33)	2022	2020-2021	45	China	Single-arm	63.8 ± 3.13	−	−			tislelizumab
W.Xing (34)	2021	2019-2020	30	China	Double-arm	63.8 ± 6.07	0.733(22/30)	−			toripalimab
N. Zhou (35)	2024	2020-2023	30	China	Single-arm	60 ± 6.0	0.833(25/30)	−	0.467(14/30)	0.533(16/30)	tislelizumab
Y. Zheng (36)	2024	2020-2021	127	China	Double-arm	66 ± 8.5	0.7638(97/127)	0.4331(55/127)	0.5512(70/127)	0.0079(1/127)	toripalimab
M. Wang (37)	2025	2021-2023	106	China	Single-arm	−	0.877(93/106)	cT1–2N+M0, or cT3–4aN0, or N+M0			camrelizumab
X. Sui (38)	2025	2022-2024	23	China	Single-arm	61.4 ± 6.1	0.478(11/23)	0.217(5/23)	0.783(18/23)	−	tislelizumab
H. Jiao (39)	2025	2022	60	China	Double-arm	62.59 ± 7.48	0.833(50/60)	0.167(10/60)	0.833(50/60)	−	nivolumab
J. Guo (40)	2025	2020-2022	29	China	Single-arm	64 ± 8.5	0.69(20/29)	0.448(13/29)	0.552(16/29)	−	sintilimab
S. Deng (41)	2025	2021-2023	42	China	Single-arm	59 ± 7.25	0.81(34/42)	0.31(13/42)	0.595(25/42)	0.095(4/42)	tislelizumab/pembrolizumab
Y. Y. Chen (42)	2024	2021-2022	37	China	Single-arm	66 ± 8.0	0.838(31/37)	T3-4NanyM0 or TanyN(+)M0			camrelizumab

SD, Standard deviation.

**Table 2:** The quality assessment of the included studies.

Study	D1	D2	D3	D4	D5	D6	D7	D8	D9	D10
D. Shen2021 (13)	1	1	1	0	1	1	1	1	0	1
Z. Wu2021 (14)	1	1	1	1	1	1	0	1	0	1
P. Yang2021 (15)	1	1	1	0	1	1	0	1	0	1
L. Zhao2021 (16)	1	1	1	0	1	0	0	1	0	1
H. Duan2022 (17)	1	1	1	0	1	0	1	1	0	1
L. Gao2022 (18)	1	1	1	0	1	1	0	1	0	1
W. He2022 (19)	1	1	1	0	1	1	1	1	0	1
B. Jiang2022 (20)	1	1	1	0	1	1	1	1	0	1
J. Liu2022 (21)	1	1	1	0	1	1	1	1	0	1
J. Liu2022 (22)	1	1	1	1	1	1	1	1	0	1
Z. Wang2022 (23)	1	1	1	0	1	0	0	1	0	1
W. Yang2022 (24)	1	1	1	0	1	1	1	1	0	1
Z. Zhang2022 (25)	1	1	1	0	1	1	1	1	0	1
X. Chen2023 (26)	1	1	1	0	1	1	0	1	0	1
J. He2023 (27)	1	1	1	0	1	0	0	1	0	1
Y. Li2023 (28)	1	1	1	0	1	1	1	1	0	1
J. Wang2024 (29)	1	1	1	0	1	0	0	1	0	1
G. Yang2023 (30)	1	1	1	0	1	1	1	1	0	1
Y. Yang2024 (31)	1	1	1	0	1	1	1	1	0	1
Z. Zhang2021 (32)	1	1	1	0	1	0	0	1	0	1
X. Yan2022 (33)	1	1	1	0	1	0	0	1	0	1
W. Xing2021 (34)	1	1	1	0	1	1	0	1	0	1
N. Zhou2024 (35)	1	1	1	0	1	1	1	1	0	1
Y. Zheng2024 (36)	1	1	1	0	1	1	1	1	0	1
Y. Y. Chen 2024 (42)	1	1	1	0	1	1	1	1	0	1
M. Wang2025 (37)	1	1	1	1	1	1	1	1	0	1
X. Sui2025 (38)	1	1	1	1	1	1	0	1	1	1
H. Jiao2025 (39)	1	1	1	1	1	1	1	1	0	1
J. Guo2025 (40)	1	1	1	0	1	1	1	1	0	1
S. Deng2025 (41)	1	1	1	1	1	1	1	1	0	1

Domains:

D1:Whether there are clear case inclusion criteria.

D2:Whether standard, credible methods have been used to identify the disease or health problem in the case.

D3:Whether effective methods are used to diagnose the disease.

D4:Whether the subjects in the case were comprehensive.

D5:Whether the inclusion of subjects in the cases was consistent.

D6:Whether the demographic information of the study subjects is clearly reported.

D7:Whether clinical information about study subjects was clearly reported.

D8:Whether the outcome of the case and follow-up results were clearly reported.

D9:Whether the geography of the case is clearly reported.

D10:Whether the statistical analysis method is appropriate.

Judgement.

1:Yes.

0:No or no information.

### 3.3 Pooled analysis of efficacy and safety-related endpoints

**Figure 2:**
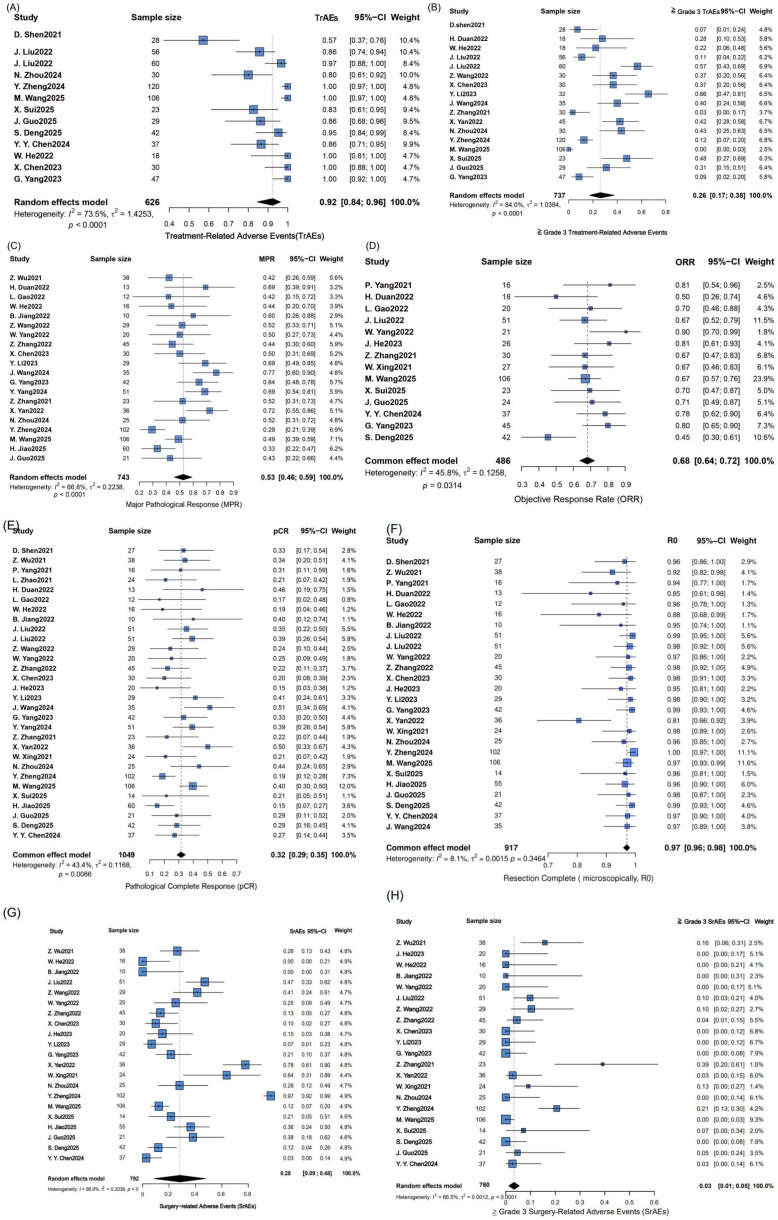
Forest plot of the efficacy and safety of neoadjuvant immunochemotherapy. This forest plot presents the pooled efficacy and safety outcomes from a meta-analysis. The MPR rate was 53%, the pCR rate was 32%, and the ORR was 68%. The *R*_0_ resection rate was high at 97%. Regarding safety, the incidence of TrAEs was 92%, while the incidence of **≥** Grade 3 TrAEs was 26%. The incidence of SrAEs was 28%, while the incidence of ≥ Grade 3 SrAEs was 3%. A random-effect model was applied to MPR, TrAEs and SrAEs outcomes due to significant heterogeneity, whereas a common-effect model was used for pCR, ORR, and *R*_0_ resection, which showed no significant heterogeneity. **(A)** TrAEs **(B)** the incidence of **≥** Grade 3 TrAEs **(C)** MPR **(D)** ORR **(E)** pCR **(F)** *R*_0_ **(G)** SrAEs **(H)** the incidence of ≥ Grade 3 SrAEs. 95%-CI, 95% confidence interval; I^2^, I- squared; τ^2^, T- squared; χ^2^, Chi- squared.

Firstly, we performed a meta-analysis of MPR rates, pCR rates, AEs, and *R*_0_ resection rates across all studies. As illustrated in **Figure 2**, in the analysis of heterogeneity, there was significant heterogeneity in the incidence of treatment-related adverse events (TrAEs) (*P* < 0.0001, I^2^ = 73.5%), the incidence of ≥ Grade 3 TrAEs (*P* < 0.0001, I^2^ = 84.0%), surgery-related adverse events (SrAEs) (*P* = 0, I^2^ = 98.9%), the incidence of ≥ Grade 3 SrAEs (*P* < 0.0001, I^2^ = 66.5%), and MPR (*P* < 0.0001, I^2^ = 66.8%), so a random effects model was adopted. There was no significant heterogeneity in pCR rates (*P* = 0.0066, I^2^ = 43.4%), ORR (*P* = 0.0314, I^2^ = 45.8%), and *R*_0_ (*P* = 0.3464, I^2^ = 8.1%), so a common effect model was used. The pooled MPR was 53% (95% confidence interval (CI): 46-59%), the pooled pCR rate was 32% (95% CI: 29-35%), and the pooled *R*_0_ rate was 97% (95% CI: 96-98%). The pooled ORR was 68% (95% CI: 64-72%). Finally, the pooled incidence of TrAEs was 92% (95% CI: 84-96%), and the pooled incidence of ≥ Grade 3 TrAEs was 26% (95% CI: 17-38%). The pooled incidence of SrAEs was 28% (95% CI: 9-48%), and the incidence of ≥ Grade 3 SrAEs was 3% (95% CI: 1-5%). In all, 9 cases of surgery-related deaths were identified. Among them, 3 cases were pulmonary complications (all related to pneumonia), 3 cases were direct surgery-related complications (hemorrhagic shock, anastomotic leakage complicated with hemorrhage, and esophagotracheal fistula, respectively), 1 case was severe infection, and 2 cases were attributed to unspecified causes (fatal due to surgery-related Grade V adverse events). (Note: TrAEs were consistently assessed during neoadjuvant immunochemotherapy (from the first dose to 7 days after the last dose) across all included studies, while SrAEs were uniformly evaluated within 30 days postoperatively). (**Supplementary Table S2**. Surgery-related adverse events (SrAEs)).

### 3.4 Exploratory subgroup analysis

**Figure 3:**
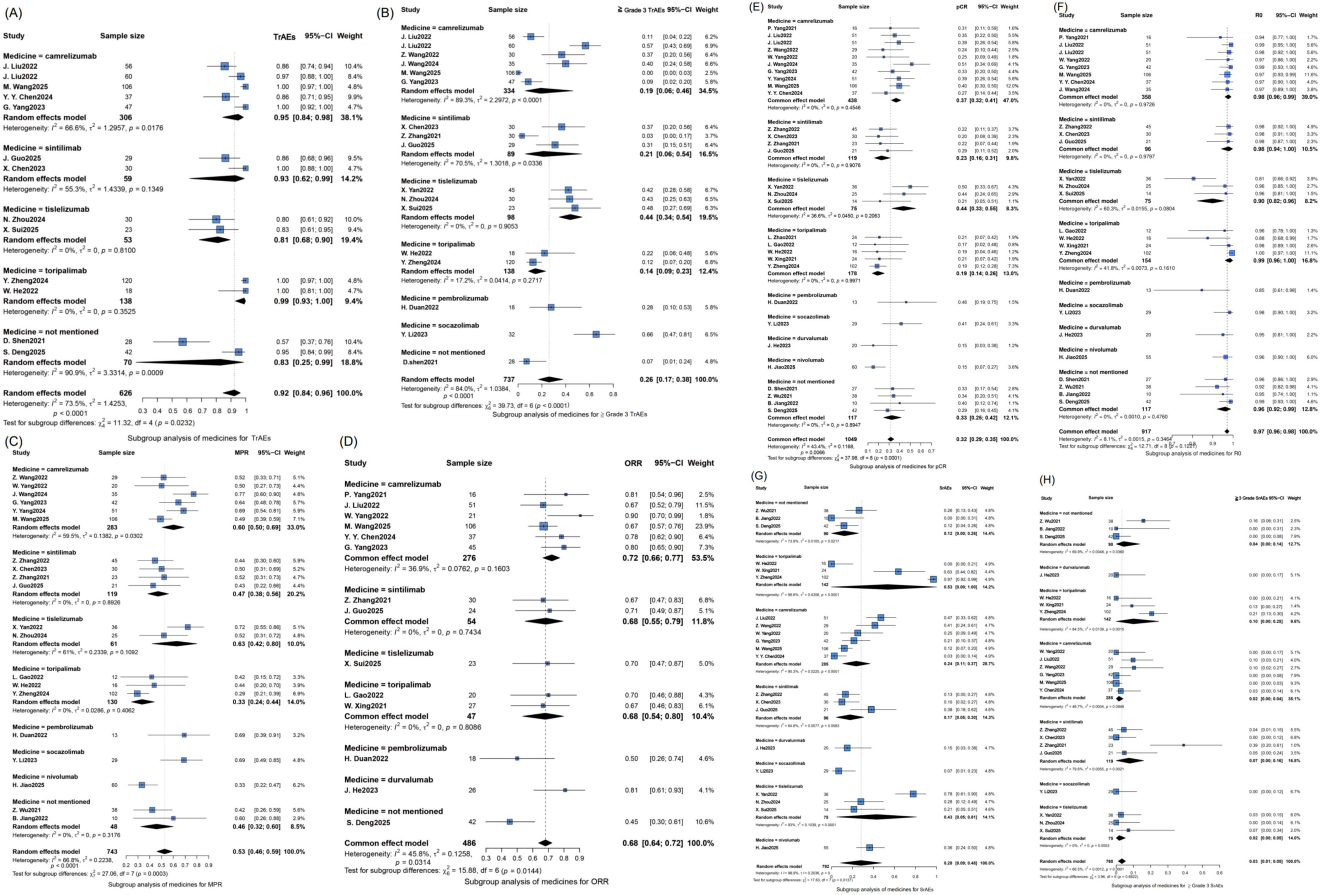
Forest plot of the efficacy and safety of subgroup analysis based on different neoadjuvant treatment types. Forest plots present pooled estimates for AEs, **≥** Grade 3 AEs, MPR, ORR, pCR, *R*_0_ resection rate, stratified by immune checkpoint inhibitor. Camrelizumab, sintilimab, and tislelizumab subgroups showed considerable heterogeneity for certain outcomes. Tislelizumab was associated with a higher incidence of **≥** Grade 3 TrAEs (pooled 44%) and MPR (pooled 63%), whereas toripalimab was associated with a lower incidence of **≥** Grade 3 TrAEs (pooled 14%) and a lower pCR rate (pooled 19%). Test for subgroup differences was significant for most outcomes, indicating the treatment effect may be modified by the specific agent used. **(A)** TrAEs **(B)** the incidence of **≥** Grade 3 TrAEs **(C)** MPR **(D)** ORR **(E)** pCR **(F)** *R*_0_ **(G)** SrAEs **(H)** the incidence of ≥ Grade 3 SrAEs. 95%-CI, 95% confidence interval; I^2^, I- squared; τ ^2^, T- squared; χ^2^, Chi- squared.

**Figure 4:**
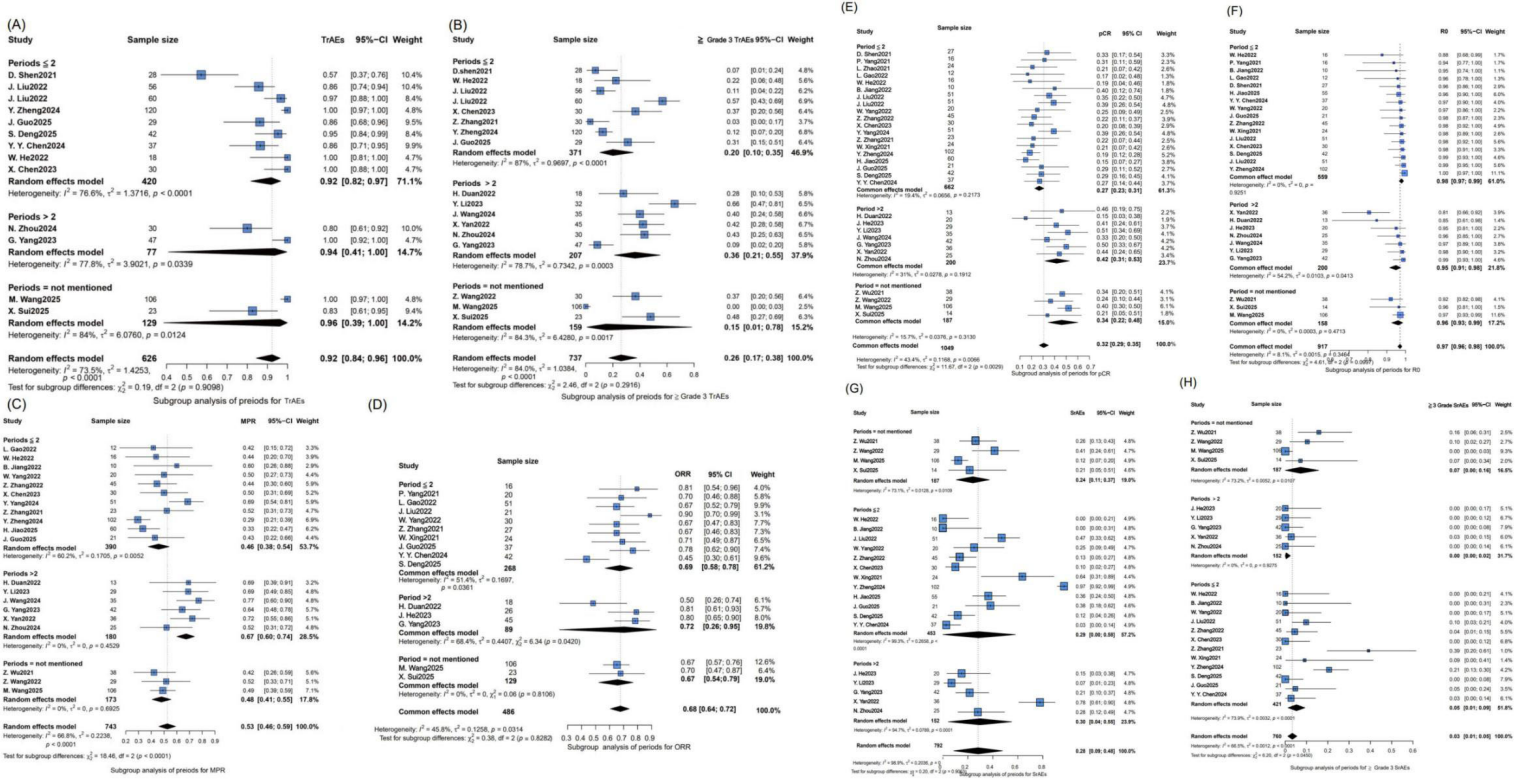
Forest plot of the efficacy and safety of subgroup analysis based on different treatment cycles. Forest plots display pooled outcomes stratified by the number of treatment cycles (≤ 2, > 2, or not specified). Extending nICT beyond 2 cycles was associated with improved pathological responses (MPR: 67% vs. 46%; pCR: 42% vs. 27%) but a higher rate of **≥** Grade 3 TrAEs (36% vs. 20%). ORR remained consistent across subgroups (67-72%), and the *R*_0_ resection rate was universally high. **(A)** TrAEs **(B)** the incidence of **≥** Grade 3 TrAEs **(C)** MPR **(D)** ORR **(E)** pCR **(F)** *R*_0_ **(G)** SrAEs **(H)** the incidence of ≥ Grade 3 SrAEs. 95%-CI, 95% confidence interval; I^2^, I- squared; τ^2^, T- squared; χ^2^, Chi- squared.

**Figure 5:**
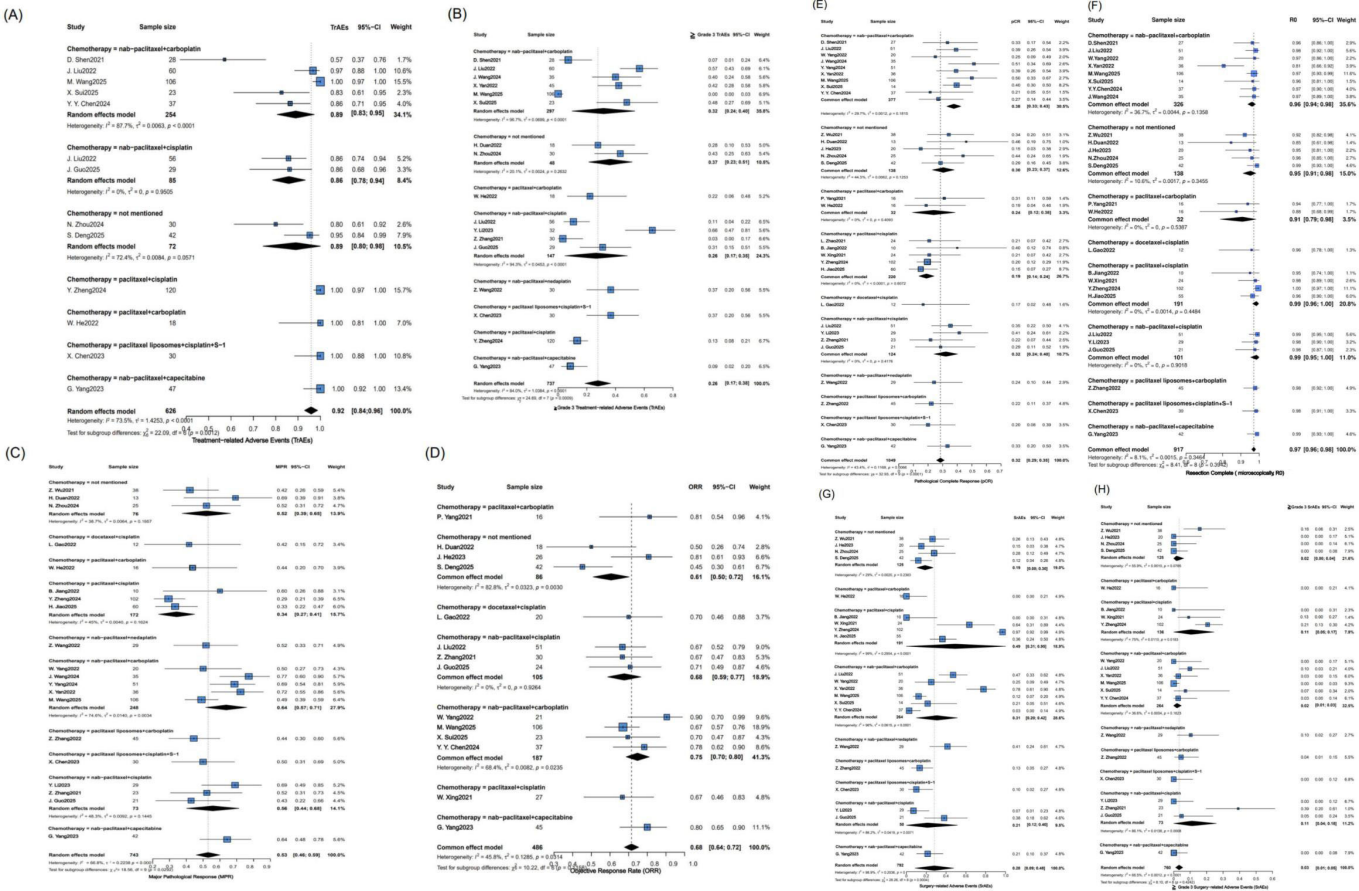
Forest plot of the efficacy and safety of subgroup analysis based on different combined chemotherapy regimens. This forest plot shows subgroup analysis of different combination chemotherapy regimens, with notable efficacy and safety differences. Nab-paclitaxel plus carboplatin showed superior efficacy. PCR was 38% (n=377) vs. 19% (n=220, paclitaxel + cisplatin); ORR was 75% (n=187) vs. 67% (n=27, paclitaxel + cisplatin); MPR was 64% (n=248) vs. 56% (n=73, nab-paclitaxel + cisplatin). R_0_ resection rate approached 1.0 uniformly across all regimens with minimal subgroup differences. The incidence of ≥ Grade 3 TrAEs was higher in nab-paclitaxel + carboplatin (32%, n=297) than nab-paclitaxel + cisplatin (26%, n=147); the incidence of ≥ Grade 3 SrAEs was 11% in both paclitaxel + cisplatin (n=136) and nab-paclitaxel + cisplatin (n=73). **(A)** TrAEs **(B)** the incidence of ≥ Grade 3 TrAEs **(C)** MPR **(D)** ORR **(E)** pCR **(F)** *R*_0_ **(G)** SrAEs **(H)** the incidence of ≥ Grade 3 SrAEs. 95%-CI, 95% confidence interval; I^2^, I- squared; τ^2^, T- squared; χ^2^, Chi- squared.

To identify potential sources of heterogeneity, exploratory subgroup analyses were further performed. The different immunotherapeutic agents, treatment periods, and combined chemotherapy regimens used in the included studies may be a source of heterogeneity. The subgroup analysis is illustrated in **Figures 3**–**5**. Based on the subgroup analysis of neoadjuvant immunotherapeutic types, tislelizumab was associated with the most favorable pathological response rates, with MPR of 63% (n = 61) and pCR of 44% (n = 75), yet it also exhibited the highest incidence of ≥ Grade 3 TrAEs (44%, n = 98). Camrelizumab demonstrated a balanced efficacy-safety profile, with MPR of 60% (n = 283), pCR of 37% (n = 438), and a lower rate of severe TrAEs (19%, n = 334). In contrast, toripalimab showed the lowest toxicity (≥ Grade 3 TrAEs: 14%, n = 138) but was less effective in terms of MPR (33%, n = 130) and pCR (19%, n = 178). The ORR was consistent across several agents, and *R*_0_ resection rates remained high across all subgroups. Based on the subgroup analysis of treatment cycles, extending neoadjuvant immunotherapy beyond two cycles was associated with significantly improved pathological outcomes, including MPR of 67% (n = 180) and pCR of 42% (n = 200), compared to 46% (n = 390) and 27% (n = 662), respectively, for shorter regimens (≤ 2 cycles). These extended neoadjuvant periods were linked to a higher incidence of ≥ Grade 3 TrAEs, with 36% (n = 207) compared with 20% (n = 371) in the shorter group. The ORR remained consistent across cycle subgroups (67–72%), and the *R*_0_ resection rate was generally high regardless of treatment duration. Regarding the subgroup analysis of different combined chemotherapy regimens, significant differences were observed in both efficacy and AEs among the regimens. In terms of efficacy, the nab-paclitaxel plus carboplatin subgroup demonstrated favorable outcomes: pCR was significantly higher than that of the paclitaxel plus cisplatin subgroup (38%, n = 377 *v.* 19%, n = 220), and ORR (75%, n = 187) also exceeded the corresponding rate in the paclitaxel plus cisplatin subgroup (67%, n = 27). Additionally, the nab-paclitaxel plus carboplatin subgroup (n = 248) achieved a higher MPR (64% *v.* 56%) than nab-paclitaxel plus cisplatin (n = 73). The *R_0_* resection rate was uniformly elevated across all regimens (predominantly approaching 1.0), with negligible variability among subgroups. Regarding AEs, the nab-paclitaxel plus carboplatin regimen (n = 297) had a higher incidence of ≥ Grade 3 TrAEs (32% *v.* 26%) than nab-paclitaxel plus cisplatin subgroup (n = 147), while the paclitaxel plus cisplatin (n = 136) and nab-paclitaxel plus cisplatin (n = 73) combinations had similar rates of ≥ Grade 3 SrAEs (both 11%).

### 3.5 Publication bias and sensitivity analyses

As displayed in **Supplementary Figure S1** and **Supplementary Table S3**, the assessment of publication bias revealed distinct patterns between efficacy and safety outcomes. Egger’s test indicated no significant bias for efficacy endpoints (ORR, pCR, MPR), while significant asymmetry was found for safety outcomes (TrAEs and ≥ Grade 3 TrAEs). Sensitivity analysis via the leave-one-out method confirmed the high robustness of all pooled estimates, as effect sizes remained stable after sequentially omitting each study. Notably, the *R*_0_ resection rate showed both robustness and zero heterogeneity. (**Supplementary Figure S2**).

## 4 Discussion

This meta-analysis evaluated the efficacy and safety of nICT in locally advanced ESCC. The pooled MPR after nICT was 53%, the pooled pCR was 32%, the pooled *R*_0_ rate was 97%, and the pooled ORR was 68%. The pooled incidence of ≥ Grade 3 TrAEs was 26%. The incidence of ≥ Grade 3 SrAEs was 3%. This study shows that nICT is effective and safe in China.

**Figure 6:**
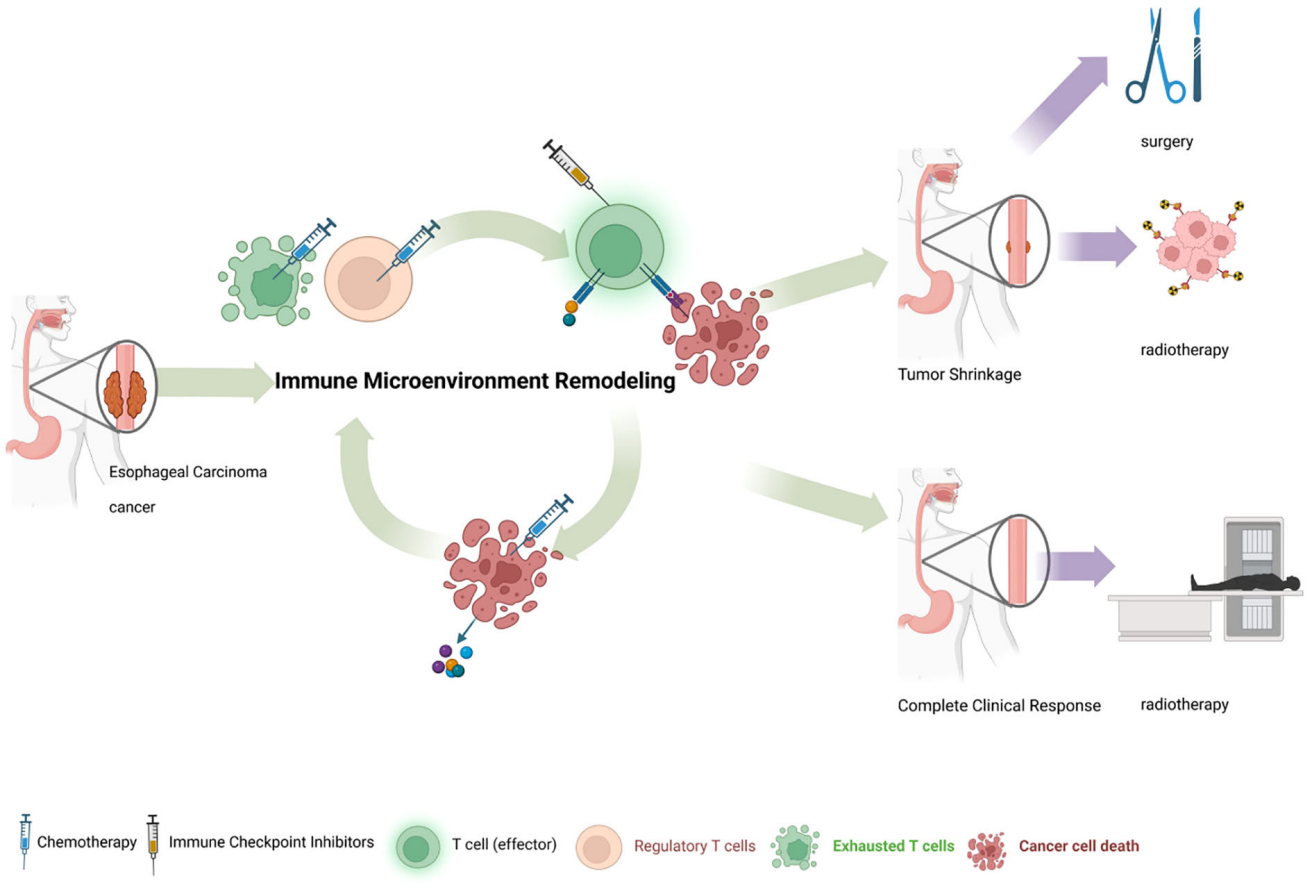
Mechanism diagram of neoadjuvant immunochemotherapy. Immunotherapy blocks the PD-1/PD-L1 signaling pathway to relieve T cell suppression and remodels the tumor immune microenvironment. Through synergistic effects with chemotherapy, this combined approach promotes significant shrinkage or disappearance of esophageal tumors, thereby creating opportunities for subsequent curative surgery, precise radiotherapy, or active surveillance strategies.

In recent years, in view of the effectiveness and safety of immunotherapy, nICT has provided new options for the treatment of locally advanced ESCC. Most clinical studies are phase I/II trials with limited enrolled patients. NICT in ESCC exerts synergistic antitumor effects through multifaceted mechanisms. Chemotherapeutic agents such as taxanes and platinum derivatives induce immunogenic cell death (ICD), releasing tumor-associated antigens and damage-associated molecular patterns (DAMPs) that promote dendritic cell maturation and subsequent cytotoxic T lymphocyte (CTL) priming (43, 44). Concurrently, immune checkpoint inhibitors (*e.g*., anti-PD-1/PD-L1 antibodies) reverse T-cell exhaustion by blocking inhibitory signals, restoring CTL proliferation, cytokine production, and tumoricidal activity (45). This combination further remodels the immunosuppressive tumor microenvironment by reducing regulatory T cells (Tregs) and myeloid-derived suppressor cells (MDSCs), while enhancing CTL infiltration and fostering long-term immune memory (46, 47) (**Figure 6**).

In this meta-analysis, the pooled rate of pCR was 32% (95% CI: 29-35%), which was significantly higher than 9% in nCT (48) and 29% in the CROSS study (5). Among the 30 studies included, 30 articles reported pCR data and 20 articles provided MPR data, and Wang (29) achieved the highest pCR and MPR of 51.4% and 77.1%, respectively. A pCR > 30% was found in 14 of the 30 articles. Interestingly, evidence showed that deferring the first dose of toripalimab to day 3, rather than administering it concurrently on day 1, may yield a higher pCR rate in chemoimmunotherapy (49). These preliminary results suggested the efficacy of nICT for achieving pathological remission. Among the 30 articles included in this review, X. Chen’s study observed a trend towards higher MPR rates in patients with PD-L1 combined positive score (CPS) ≥ 1 (66.7% *v*. 37.5%; *P* = 0.23) (26). X. Yan’s results indicated that tumor proportion score (TPS) levels in both MPR and pCR groups were significantly higher than those in non-MPR (*P* = 0.034) and non-pCR groups (*P* = 0.011), respectively (33). However, no significant differences in PD-L1 CPS were observed between MPR and non-MPR groups (*P* = 0.66), or between pCR and non-pCR groups (*P* = 0.6) (33). Additionally, J. Wang’s study identified PD-L1 as an independent risk factor for pCR (29); however, no correlation was established between PD-L1 expression and pathological regression, nor was any subgroup analysis performed in other reported investigations. Due to the limited follow-up time of the included studies, few data related to long-term survival were extracted. The 1-year OS rate after receiving nICT was 86.0-97.3% (35, 42). Long-term survival data are not yet mature, and several large-scale randomized controlled trials are underway. Nevertheless, a retrospective study indicated that patients with locally advanced ESCC receiving nICT demonstrate superior overall and disease-free survival compared to nCRT, primarily through reduced incidence of distant metastasis (50). The pooled ORR of the study was 68%, which indicated that the neoadjuvant immunotherapy improved the efficacy. The ESCORT-NEO study (51), announced at the ASCO meeting in 2024, represents the first phase III randomized controlled trial of nICT in the perioperative phase of ESCC. Some 391 patients were randomly assigned to neoadjuvant camrelizumab plus chemotherapy or nCT in three groups. This study implies that nICT demonstrates a better pCR and tolerable safety profile than nCT in patients with locally resectable ESCC. The results of the Neotorch study also support the data in our meta-analysis (36).

As the rate of pCR increases, we have also had more discussions regarding treatment plans subsequent to neoadjuvant therapy. The results of the retrospective study indicated that although surgery can reduce the risk of recurrence, it does not significantly improve the overall long-term survival rate (52). The SANO trial demonstrated that active surveillance is non-inferior to surgery in specific patient populations, although organ-preserving treatment currently remains at an investigative stage and requires careful patient selection (53). The compelling efficacy of nICT, as definitively established by the ESCORT-NEO trial, has set a new benchmark in the management of resectable ESCC. This paradigm shift naturally prompts the investigation of whether the integration of immune checkpoint inhibitors into an alternative, and potentially more potent, neoadjuvant backbone could yield even greater therapeutic benefits. In this context, the recent phase II EC-CRT-001 study offers pivotal and encouraging data. This trial evaluated sintilimab in combination with concurrent nCRT, reporting a remarkable pCR of 50.0% and MPR of 77.8% (54). The treatment regimen exhibited a manageable safety profile, with no unexpected toxicities arising from the combination of immunotherapy and radiation. More neoadjuvant treatment modalities – neoadjuvant immunotherapy combined with radiotherapy and chemotherapy, neoadjuvant immunotherapy combined with targeted therapy, *etc.*, – are currently used (**Table 3**).

**Table 3:** The ongoing perioperative study on esophageal squamous cell carcinoma.

ID	Country	Study design	Clinical stages	Treatment	Immunotherapy	Chemotherapy	Primary endpoint	Secondary endpoint
NCT06509568	China	a multicenter, prospective, randomized phase II trial	cT3-4aN0 or T2-4aN+	neoadjuvant chemoradiotherapy combined with immunotherapy (nCRIT) vs. nICT	tislelizumab	paclitaxel (albumin-bound) + carboplatin	pCR	R0 resection rate,MPR, 2-year EFS, 2-year OS, adverse events and HRQoL
NCT06907602	China	a multicenter, prospective, randomized phase II trial	T3 or resectable T4, N0 or N+,M0 or M1a	nCRIT vs. nICT (2 cycles) vs. nCIT (4 cycles)	pembrolizumab	carboplatin + nab-paclitaxel	pathological response and 2-year and 5-year OS	adverse effect incidence, R0 resection rate, postoperative complications and 2-year and 5-year DFS
NCT04807673	China	a multicenter randomized, controlled phase III trial	cT1-3N1-2M0, cT2-3N0M0	nCRIT vs. nCRT	pembrolizumab	paclitaxel + cisplatin	EFS	OS, DFS, MPR, ORR, pCR, assessment in perioperation, incidence of treatment-emergent adverse events, quality of life differences (EORTC QLQ-OES18) and quality of life differences (EORTC QLQ-C30)
NCT06385730	China	a non-randomized phase II trial	T1N1-3M0 or T2-3N0-3M0 (M1 lymph node metastasis confined to the supraclavicular lymph nodes)	neoadjuvant anti-PD-1 vs. neoadjuvant anti-PD-1 with LDRT	toripalimab	none	MPR	pCR, adverse events and treatment-related adverse events, R0 resection rate, ORR, EFS,OS and correlation between potential biomarkers and tumor response
NCT06601309	China	an exploratory phase II single-arm study	clinical stage II-III according to the AJCC/UICC 8th Edition	neoadjuvant immunotherapy (CPS ≥ 20), nICT (CPS 10-20), nCRT (CPS < 10)	serplulimab	paclitaxel + cisplatin	pCR	R0 resection rate, treatment-related toxicity, DFS, TMB, MSI, ctDNA, andMPR
NCT06843889	China	a phase II single-arm study	T2-4a, N0-3, M0	Phase 1: toripalimab + investigator’s choice of clinical conventional chemotherapy; Phase 2: toripalimab + radiotherapy	toripalimab	investigator’s choice of clinical conventional chemotherapy	pCR	MPR, DFS, OS and adverse events,
NCT06426797	China	a phase II single-arm study	cT1-2N1-3M0, T2 (diameter≥3 cm) N0M0 or T3-4aN0-3M0 (stage II-IVA)	cadonilimab + anlotinib	cadonilimab	none	pCR	MPR, ORR, treatment emergent adverse events and EFS
NCT04973306	China	a multi-center prospective randomized clinical trial	clinical II-III (AJCC/UICC 8th Edition)	nCRIT vs. nCRT	tislelizumab	carboplatin + paclitaxel	pCR, OS	treatment related complications, PFS, R0 resection rate, number and location of positive lymph nodes, overall quality of life, quality of life in eating and swallowing function, correlation between genetic profile and tumor response and RFS
NCT05743504	China	a phase Ib/II single-arm trial	T1-2N2-3M0 or T3N1-3M0	nCRIT	tiragolumab, atezolizumab	paclitaxel + cisplatin	pCR	side effect evaluation, number of participants with treatment-related adverse events as assessed by CTCAE v5.0, number of participants with surgical complications and number of participants with delay in planned radical esophagectomy
NCT06446726	China	a randomized clinical trial	cT1b-cT2 N1–2 M0 or cT3-cT4a N0–2 M0 (AJCC/UICC 8th Edition)	nICT+Low-dose radiotherapy (4Gy/2f vs. 6Gy/3f vs. 8Gy/4f)	tislelizumab	nab-paclitaxel + cisplatin	pCR	R0 resection rate, 1/2 year EFS and ORR
NCT04229459	Israel	a phase II single-arm study	cT3NxM0, TxN1M0	chemotherapy and chemoradiation combined with cetuximab followed by nivolumab and cetuximab as neoadjuvant treatment	nivolumab	cisplatin + 5-FU	pCR, PFS and incidence of treatment-emergent adverse events	OS
NCT06354530	China	a single-center, open-label, non-inferior, randomized controlled, interventional study	cT1-4aN+M0 or cT3-4aN0M0	nICT + anlotinib vs. nCRIT	camrelizumab	paclitaxel/nab-paclitaxel + cisplatin/carboplatin	pCR, MPR	ORR, 3-year DFS, 3-year OS and R0 resection rate
NCT05244798	China	a multicenter, randomized, controlled, phase III trial	cT1N2-3M0 or cT2-4aN0-3M0(8th UICC-TNM stage)	nCRIT vs. nICT vs. nCRT	sintilimab	albumin-paclitaxel + carboplatin	pCR	treatment related adverse event, MPR, R0 resection rate,EFS, OS, DFS, DCR, ORR and ctDNA
NCT06814158	China	a phase II single-arm study	cT1N2M0, cT2-3N0-2M0 (stage II/III)	ivonescimab + chemotherapy	ivonescimab	cisplatin + paclitaxel	pCR	
NCT06056336	China	a prospective single-arm, phase II Study	cT1b-3N1-3M0 or T3N0M0	nICT	tislelizumab	nab-paclitaxel + carboplatin	2-year DFS in non-pCR patients	2-year DFS in pCR patients, pCR, MPR, R0 resection rate, adverse events and OS
NCT06403878	China	a prospective, single-arm, phase II study	T2-4aN0M0 or T1-4aN+M0	nICT	sintilimab	nab-paclitaxel + carboplatin	pCR	1-year DFS, surgical completion rate, R0 resection rate and incidence of adverse events to neoadjuvant and surgical treatment
NCT05821452	China	a phase II, prospective, two-arm clinical study	cT4 or at least one group of lymph nodes invade the surrounding organs and unresectable lymph nodes	nICT vs. nCRT	camrelizumab	paclitaxel + cisplatin	R0 removal rate, MPR	PFS, OS

The above studies were all sourced from ClinicalTrials.gov up to 2025-10-02.

In terms of surgery-related events, the *R*_0_ resection rate is an important indicator used to evaluate the surgical effect of ESCC, and a higher *R*_0_ represents a better prognosis (55). The pooled *R*_0_ value in the present meta-analysis was 97%. Among the 26 reports that provided *R*_0_ data, 12 studies achieved a 100% *R*_0_ rate. The mean operation time after nICT ranged from 194 to 362 minutes, and the mean intraoperative blood loss was 131–212 mL. These results indicated that neoadjuvant immunotherapy did not increase the difficulty of surgical resection and associated risks.

Our analysis also focused on the safety of nICT: 17 articles reported the incidence of ≥ Grade 3 TrAEs, with a pooled incidence of 26%, which was lower than the 34.1% found in the ESCORT-NEO study (51). The main TrAEs in hematological toxicity included leukopenia, neutropenia, and thrombocytopenia. Non-hematological toxicity AEs primarily comprised nausea, vomiting, fatigue, loss of appetite, and rash. Postoperative complications were mostly pneumonia and anastomotic fistula. One patient underwent reoperation due to poor control of postoperative complications (34). Fatal surgical complications were rare, and 9 cases of surgery-related deaths were identified. These included pulmonary-related complications (1 case each in H. Duan 2022 (17), W. Xing 2021 (34) and Y. Y. Chen 2024 (42), all related to pneumonia), direct surgical complications (1 case each of hemorrhagic shock in Z. Zhang 2022 (25), anastomotic leakage with hemorrhage in X. Yan 2022 (33), and esophagotracheal fistula in X. Sui 2025 (38)), severe infection (1 case in H. Jiao 2025 (39)), and unspecified causes (2 cases in Y. Zheng 2024 (36), fatal due to surgery-related Grade V adverse events). Regarding immune-related adverse events (ir-AEs), J. Liu’s results reported Grade 1–2 events in 21 patients (37.5%), while ≥ Grade 3 events occurred in two patients (3.6%) (21). The three most common adverse effects were maculopapular rash, reactive cutaneous capillary endothelial hyperplasia (RCCEP), and nausea, with incidence rates of 12.5%, 8.9%, and 7.1% respectively (21). L. Gao’s paper documented two cases of immune-related dermatitis, accounting for 10% (18). W. Xing’s report identified one case each of immune-mediated colitis and myocarditis (34), whereas Y. Li (in 2023) and G. Yang (also in 2023) reported the highest incidences of immune-related hyperthyroidism at 6.3% and 8.5%, respectively (28, 30). H. Duan’s research in 2022 implied that thyroid dysfunction, rash, and pneumonia may be associated with immunotherapy (17). Many clinical guidelines have provided guidelines for handling ir-AEs based on the patient’s symptoms and test results. Mild cases will be observed, while severe cases will be treated with oral corticosteroids and intravenous hormone therapy. Usually, ir-AEs are reversible; however, severe ir-AEs can lead to discontinuation of immunotherapy or even death (56).

According to the results of our subgroup analysis, patients receiving more than two cycles of neoadjuvant therapy exhibited higher pCR and MPR rates; however, this conclusion requires further validation in prospective studies (37). This highlighted the importance of personalized treatment plans based on patient tolerance and imaging manifestations during neoadjuvant therapy. It is necessary to discover (and validate) predictive biomarkers that can be used to forecast therapeutic efficacy and guide clinical decision-making. The heterogeneity in treatment outcomes underscores the limitation of PD-L1 as a standalone predictor, as responses occur across all levels of expression (including low or undetectable PD-L1 expression) despite trends favoring PD-L1 positive patients in trials such as PEN-ICE and KEEP-G 03 (17, 26). Circulating tumor DNA (ctDNA) clearance following neoadjuvant therapy has emerged as a powerful prognostic tool, strongly correlating with pathological response and recurrence-free survival, with preoperative ctDNA positivity serving as an independent prognostic factor for inferior disease-free survival (39). Meanwhile, ctDNA plays a crucial role in guiding organ preservation screening and treatment decision-making (57). The composition and dynamics of specific immune cells within the tumor microenvironment, such as T cells and macrophage subsets, are critical determinants of treatment response and survival in ESCC (14, 17, 19, 24, 58). Furthermore, distinct molecular markers, including ORMDL1 overexpression, are associated with disease progression and prognosis through their modulation of the immune landscape (59). These results indicate that no single biomarker is sufficient; future strategies must rely on integrated models that synergize dynamic, cellular, and molecular data to enable precision therapy.

## 5 Conclusion

This meta-analysis indicated that nICT is non-inferior to nCRT in terms of pCR, MPR, and *R*_0_ resection rates in China. Furthermore, this combination was tolerable and safe without unexpected increase in toxicity. In conclusion, the results of this comprehensive meta-analysis suggested nICT as an optimal future treatment strategy. This study integrates existing evidence from studies on nICT and, in the absence of higher-level phase III randomized controlled trials, provides a comprehensive and effective analysis of the efficacy and safety of nICT in locally advanced ESCC. It also offers objective evidence for future related studies. If nICT demonstrates efficacy equivalent to standard nCRT, immunotherapy holds advantages over radiotherapy in terms of convenience and timeliness, making it more acceptable to patients. Furthermore, identifying predictive biomarkers or developing efficacy prediction models will help precisely select patients most likely to benefit from neoadjuvant immunotherapy. The emergence of more phase III studies in the future will provide higher-level evidence for nICT, potentially leading to changes in clinical practice.

## 6 Limitations

There are several limitations in this meta-analysis. Firstly, the geographical restriction of our evidence base to China represents a key limitation. Almost all the included articles were single-arm trials, which were affected by enrolment factors (including age, gender, tumor stage, *etc.*). In the comparative analysis, data from previously published studies on nCT and nCRT were compared, and the results represented a non-contemporaneous comparison. Secondly, the total sample sizes were limited, coupled with a short follow-up period, compromising the generalizability and reliability of the findings. Furthermore, the lack of survival data made it difficult to prove the long-term effectiveness of neoadjuvant immunotherapy. Therefore, whether the favorable pCR and MPR rates observed in the present meta-analysis will translate into improving OS outcomes warrants the implementation of further evidence-based studies. Ultimately, this study lacked sufficient evidence to assess the correlation between PD-L1 expression and pathological regression. It is anticipated that future research will increasingly focus on identifying biomarkers related to the efficacy of immunotherapy.

## Data Availability

The original contributions presented in the study are included in the article/**Supplementary Material**. Further inquiries can be directed to the corresponding author/s.
