# Supplementary material for: The safety and efficacy of neoadjuvant immunochemotherapy in locally advanced esophageal squamous cell carcinoma: a meta-analysis and systematic review
Source: Front Immunol. 2026 Feb 10;17:1687326. doi: 10.3389/fimmu.2026.1687326 (PMC12929401; doi:10.3389/fimmu.2026.1687326)
Supplement: Supplementary file 1 [file DataSheet1.docx]

Supplementary Material

# Supplementary Figures and Tables

## Supplementary Figures


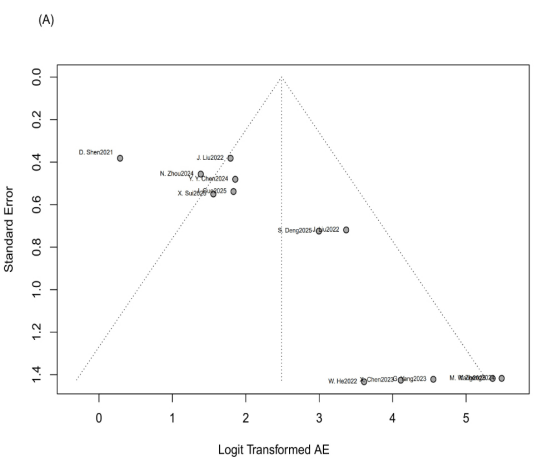

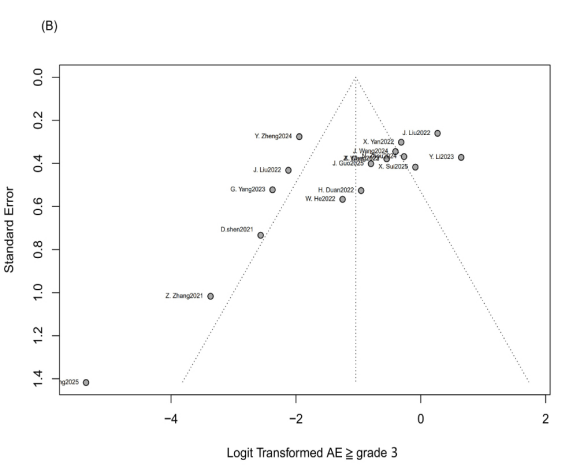


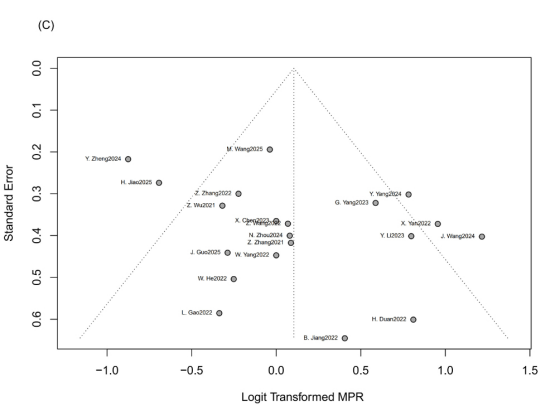

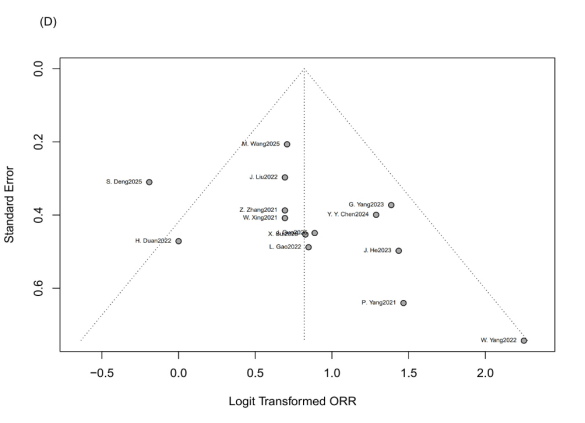


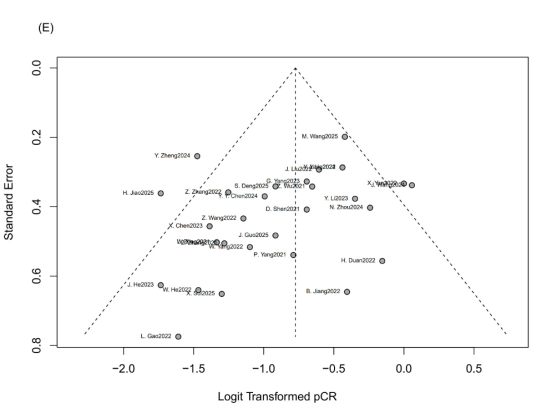

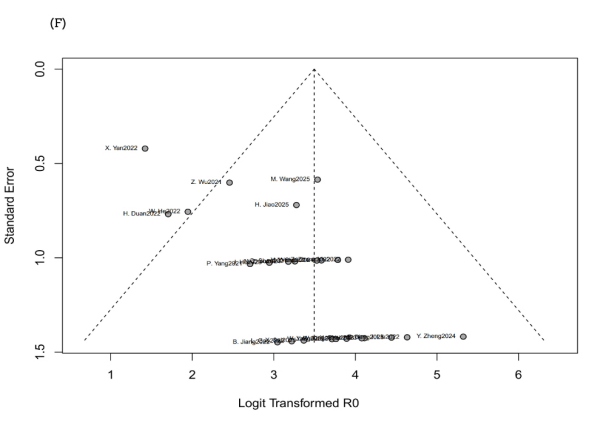


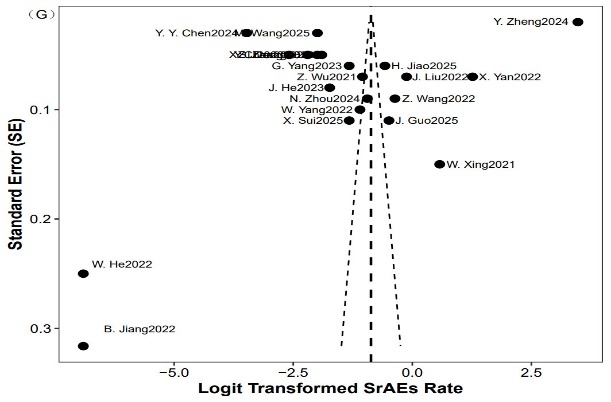

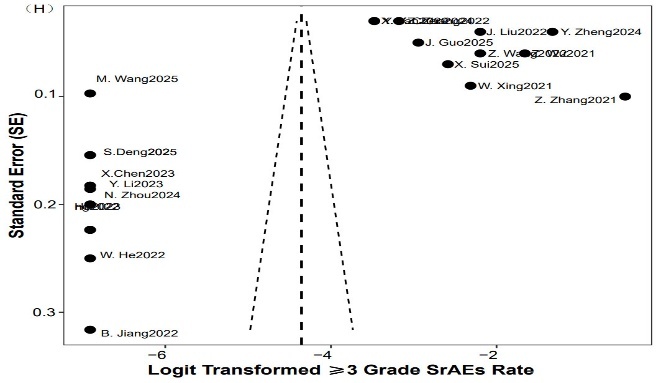


**Supplementary Figure 1.** Funnel plots for publication bias


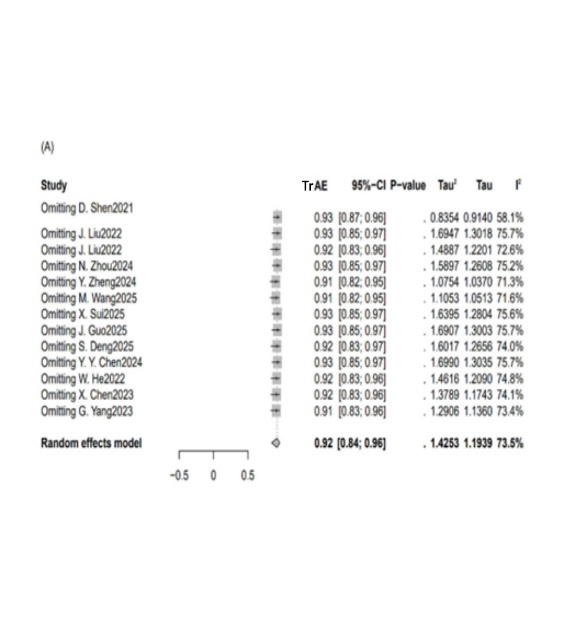

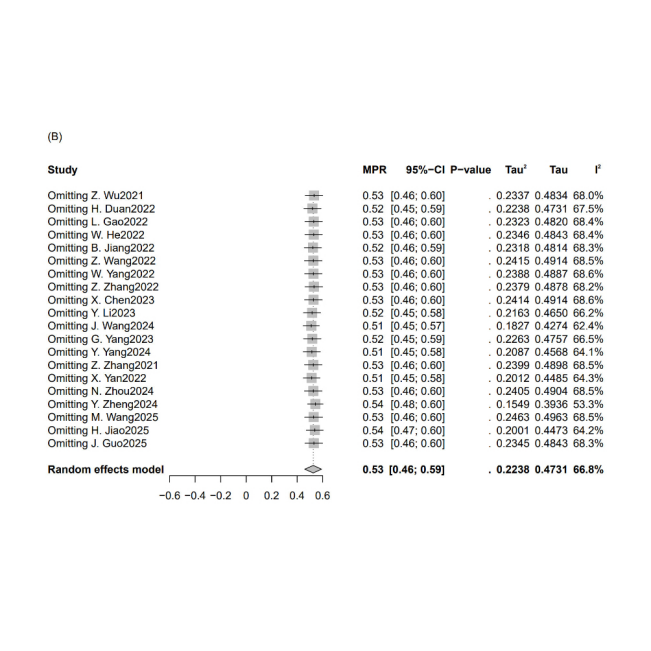


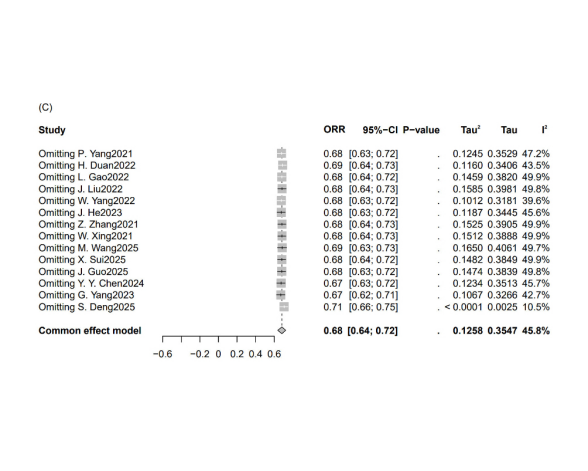

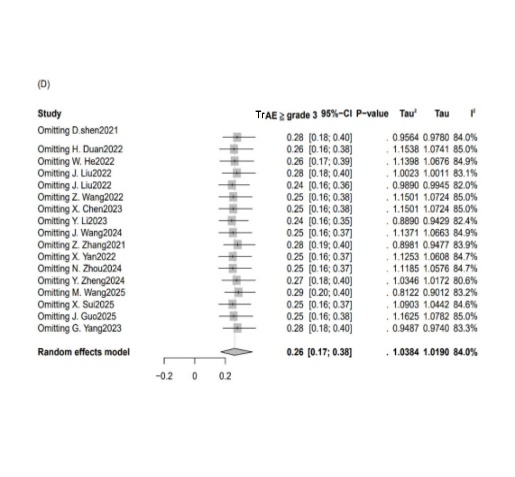


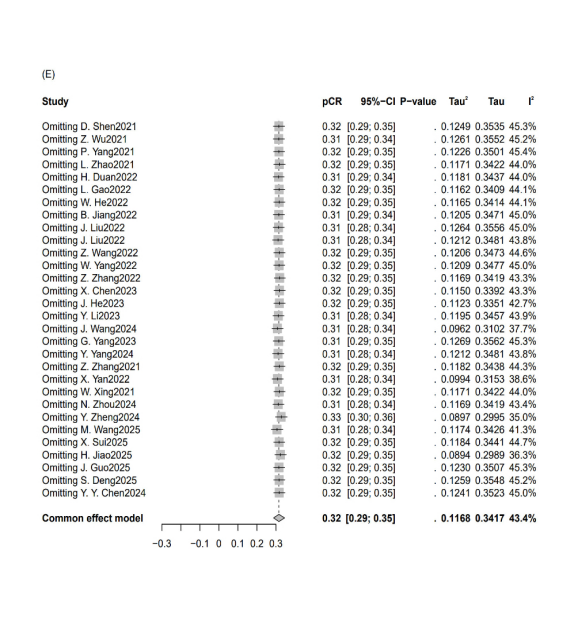

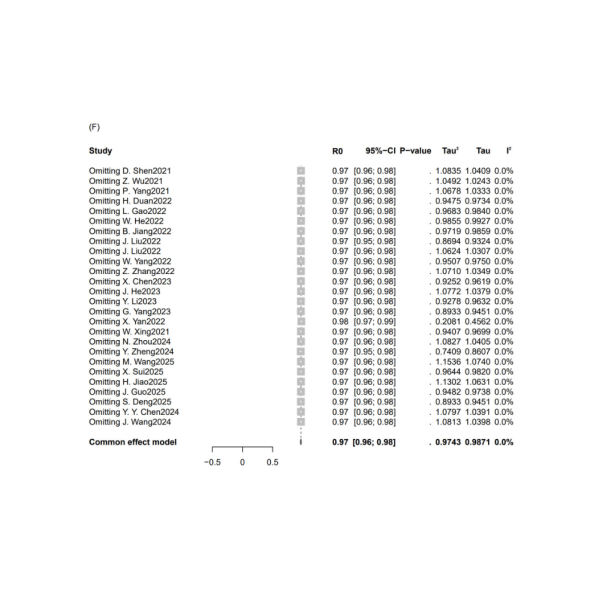


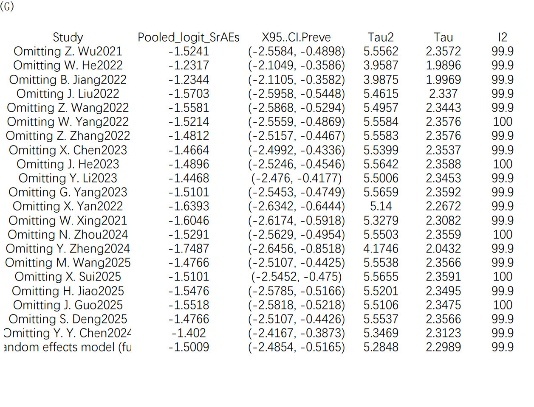

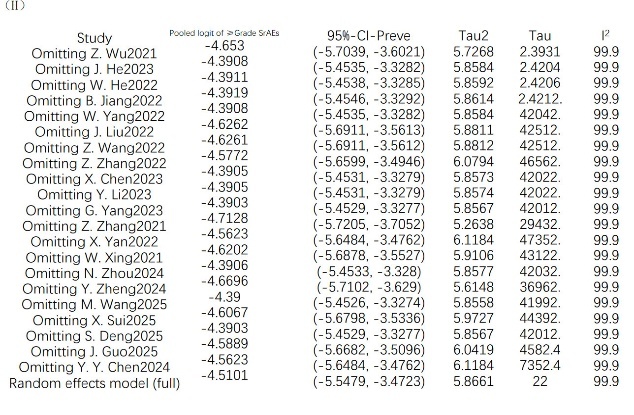


**Supplementary Figure 2.** Leave-one-out plots for sensitivity analyses

## Supplementary Tables

**Supplementary Table1.** Search strategies

Search Date: June 30, 2025
Search Concept: Neoadjuvant Immunotherapy for Esophageal Cancer

| **Database** | **Platform** | **Search Results** | **Search Strategy** |
| --- | --- | --- | --- |
| PubMed | NCBI | 415 | #1 (Immunotherapy[MeSH Terms] OR Immunotherapy[tiab] OR Immunotherapies[tiab])  #2 (Neoadjuvant Therapy[MeSH Terms] OR "Neoadjuvant Therapy"[tiab] OR "Neoadjuvant Therapies"[tiab] OR "Therapy, Neoadjuvant"[tiab] OR "Neoadjuvant Treatment"[tiab] OR "Neoadjuvant Treatments"[tiab] OR "Treatment, Neoadjuvant"[tiab] OR "Neoadjuvant Chemoradiotherapy"[tiab] OR "Chemoradiotherapy, Neoadjuvant"[tiab] OR "Neoadjuvant Chemoradiotherapies"[tiab] OR "Neoadjuvant Chemoradiation Therapy"[tiab] OR "Chemoradiation Therapy, Neoadjuvant"[tiab] OR "Neoadjuvant Chemoradiation Therapies"[tiab] OR "Therapy, Neoadjuvant Chemoradiation"[tiab] OR "Neoadjuvant Chemoradiation Treatment"[tiab] OR "Chemoradiation Treatment, Neoadjuvant"[tiab] OR "Neoadjuvant Chemoradiation Treatments"[tiab] OR "Treatment, Neoadjuvant Chemoradiation"[tiab] OR "Neoadjuvant Chemoradiation"[tiab] OR "Chemoradiation, Neoadjuvant"[tiab] OR "Neoadjuvant Chemoradiations"[tiab] OR "Neoadjuvant Radiotherapy"[tiab] OR "Neoadjuvant Radiotherapies"[tiab] OR "Radiotherapy, Neoadjuvant"[tiab] OR "Neoadjuvant Radiation Treatment"[tiab] OR "Neoadjuvant Radiation Treatments"[tiab] OR "Radiation Treatment, Neoadjuvant"[tiab] OR "Treatment, Neoadjuvant Radiation"[tiab] OR "Neoadjuvant Radiation Therapy"[tiab] OR "Neoadjuvant Radiation Therapies"[tiab] OR "Radiation Therapy, Neoadjuvant"[tiab] OR "Therapy, Neoadjuvant Radiation"[tiab] OR "Neoadjuvant Radiation"[tiab] OR "Neoadjuvant Radiations"[tiab] OR "Radiation, Neoadjuvant"[tiab] OR "Neoadjuvant Chemotherapy"[tiab] OR "Chemotherapy, Neoadjuvant"[tiab] OR "Neoadjuvant Chemotherapies"[tiab] OR "Neoadjuvant Chemotherapy Treatment"[tiab] OR "Chemotherapy Treatment, Neoadjuvant"[tiab] OR "Neoadjuvant Chemotherapy Treatments"[tiab] OR "Treatment, Neoadjuvant Chemotherapy"[tiab] OR "Neoadjuvant Systemic Therapy"[tiab] OR "Neoadjuvant Systemic Therapies"[tiab] OR "Systemic Therapy, Neoadjuvant"[tiab] OR "Neoadjuvant Systemic Treatment"[tiab] OR "Neoadjuvant Systemic Treatments"[tiab] OR "Systemic Treatment, Neoadjuvant"[tiab] OR "Treatment, Neoadjuvant Systemic"[tiab]) #3 (Esophageal Neoplasms[MeSH Terms] OR "Esophageal Neoplasm"[tiab] OR "Neoplasm, Esophageal"[tiab] OR "Esophagus Neoplasm"[tiab] OR "Esophagus Neoplasms"[tiab] OR "Neoplasm, Esophagus"[tiab] OR "Neoplasms, Esophagus"[tiab] OR "Neoplasms, Esophageal"[tiab] OR "Cancer of Esophagus"[tiab] OR "Cancer of the Esophagus"[tiab] OR "Esophagus Cancer"[tiab] OR "Esophageal Cancer"[tiab]) #4 #1 AND #2 AND #3 |
| Embase | Ovid | 153 | 1. exp Immunotherapy/ OR Immunotherap*.ti,ab. 2. exp neoadjuvant therapy/ OR ("Neoadjuvant Therapy" OR "Neoadjuvant Therapies" OR "Therapy, Neoadjuvant" OR "Neoadjuvant Treatment" OR "Neoadjuvant Treatments" OR "Treatment, Neoadjuvant" OR "Neoadjuvant Chemoradiotherapy" OR "Chemoradiotherapy, Neoadjuvant" OR "Neoadjuvant Chemoradiation" OR "Neoadjuvant Radiotherapy" OR "Neoadjuvant Chemotherapy" OR "Neoadjuvant Systemic Therapy").ti,ab. 3. exp esophagus tumor/ OR ("Esophageal Neoplasms" OR "Esophageal Cancer" OR "Esophagus Cancer" OR "Cancer of Esophagus").ti,ab. 4. 1 AND 2 AND 3 |
| Web of Science | Clarivate | 473 | #1 TS=(Immunotherapy OR Immunotherapies) #2 TS=("Neoadjuvant Therapy" OR "Neoadjuvant Therapies" OR "Neoadjuvant Treatment" OR "Neoadjuvant Chemoradiotherapy" OR "Neoadjuvant Chemoradiation" OR "Neoadjuvant Radiotherapy" OR "Neoadjuvant Chemotherapy" OR "Neoadjuvant Systemic Therapy") #3 TS=("Esophageal Neoplasms" OR "Esophageal Cancer" OR "Esophagus Cancer" OR "Cancer of Esophagus") #4 #3 AND #2 AND #1 |
| Cochrane Library | Wiley | 57 | #1 [mh Immunotherapy] OR (Immunotherapy OR Immunotherapies):ti,ab,kw #2 [mh "Neoadjuvant Therapy"] OR ("Neoadjuvant Therapy" OR "Neoadjuvant Therapies" OR "Neoadjuvant Treatment" OR "Neoadjuvant Chemoradiotherapy" OR "Neoadjuvant Chemoradiation" OR "Neoadjuvant Radiotherapy" OR "Neoadjuvant Chemotherapy" OR "Neoadjuvant Systemic Therapy"):ti,ab,kw #3 [mh "Esophageal Neoplasms"] OR ("Esophageal Neoplasms" OR "Esophageal Cancer" OR "Esophagus Cancer"):ti,ab,kw #4 #1 AND #2 AND #3 Searched in: Cochrane Central Register of Controlled Trials (CENTRAL) |
| Total (before deduplication) |  | 1098 |  |

**Note:**The search strategy was designed to be comprehensive and included a wide range of synonyms and related terms for the key concepts. The PubMed strategy is shown in its full detail, while strategies for other databases were adapted using the respective platform's syntax and controlled vocabulary (e.g., Emtree in Embase). No date or language filters were applied.

**Supplementary Table 2.** Surgery-related adverse events (SrAEs)

| Study | Total Incidence Rate of Adverse Reactions | Individual Complications (Type/Number of Cases/Incidence Rate) | Grade 3 or Higher Adverse Reactions |
| --- | --- | --- | --- |
| D. Shen2021 | Not specified (only individual statistics) | Anastomotic leakage/5 cases/18.5%; Pleural effusion/4 cases/14.8%; Pulmonary complications (including pneumonia)/3 cases/11.1%; Chylothorax, recurrent laryngeal nerve palsy, atrial fibrillation, hematological-related complications/2 cases each/7.4%; Ascites/1 case/3.7% | None; No intraoperative complications or in-hospital deaths; all surgery-related adverse reactions were postoperative non-fatal complications |
| Z. Wu2021 | 26.32% (10/38) | Pneumonia/9 cases; Chylothorax/1 case; Wound infection/1 case; Reoperation required/1 case; Reintubation required/5 cases (complications corresponding to grades not listed separately) | Present (6 cases, 15.79%); Grade 3a: 1 case (2.63%), Grade 4a: 5 cases (13.16%); no Grade 5 (death) cases; Grade 3a: severe complications requiring medical intervention or minimally invasive treatment; Grade 4a: life-threatening complications requiring ICU admission (all 5 Grade 4a patients admitted to ICU as specified in the original text) |
| H. Duan2022 | Total incidence rate not calculated (due to presence of combined complications) | Hoarseness (recurrent laryngeal nerve palsy)/5 cases/38.5%; Pneumonia/4 cases/30.8%; Empyema/3 cases/23.1%; Atelectasis/2 cases/15.4%; Heart failure/2 cases/15.4%; Respiratory failure/1 case/7.7%; Anastomotic leakage/1 case/7.7% | Present (6 cases, including 1 death); Grade 4 respiratory failure (1 case, life-threatening requiring mechanical ventilation support), Grade 3 heart failure (2 cases, requiring medical intervention or ICU monitoring), Grade 3 empyema (3 cases, requiring thoracic drainage + antibiotic treatment); Grade 5 death (1 case, 7.7%): Patient P11 developed pneumonia + immune-related pneumonia 1 day postoperatively, condition deteriorated despite treatment, and died 22 days postoperatively (related to the combination of surgical complications + immune therapy-related pneumonia, with surgery-related pneumonia as the direct cause) |
| L. Gao2022 | Total incidence rate not calculated (possible combined complications) | Pneumonia/3 cases/25.0%; Cardiac events (specific type not specified)/2 cases/16.7%; Chylothorax/1 case/8.3%; Anastomotic leakage/1 case/8.3% | None; No 30-day mortality or readmission cases; all postoperative complications were described as "manageable" without life-threatening (Grade 3 or higher) records |
| W. He2022 | 0% | No surgery-related adverse reactions (including anastomotic leakage, pneumonia, chylothorax, etc.) | None; Grade 3 or higher treatment-related AEs (e.g., neutropenia, fatigue) were from the neoadjuvant therapy phase (chemotherapy + immunotherapy) and unrelated to surgical procedures |
| B. Jiang2022 | 0% | No surgery-related adverse reactions | None; No Grade 3-4 events or treatment-related deaths |
| J. Liu2022 | 47.1% (24/51) | Pulmonary complications (pneumonia/respiratory failure/pleural effusion/pneumothorax)/9 cases/17.6%; Severe cardiac complications (supraventricular tachycardia/congestive heart failure)/2 cases/3.9%; Anastomotic leakage/5 cases/9.8%; Recurrent laryngeal nerve palsy/13 cases/25.5%; Chylothorax/4 cases/7.8% | Present (5 cases, 9.8%); Grade 4 respiratory failure (1 case, requiring respiratory support), Grade 3 anastomotic leakage (3 cases), Grade 3 chylothorax (1 case, requiring interventional or surgical treatment), Grade 3 recurrent laryngeal nerve palsy (1 case, requiring elective surgery such as tracheotomy) |
| Z. Wang2022 | 41.4% (12/29) | Pneumonia/2 cases/6.9%; Recurrent laryngeal nerve palsy/2 cases/6.9%; Pleural effusion (including 3 Grade IIIa cases)/6 cases/20.7%; Wound infection/2 cases/6.9%; Anastomotic leakage/3 cases/10.3%; Arrhythmia/3 cases/10.3% | Present (3 cases, 10.3%); Grade IIIa pleural effusion (3 cases, requiring medical intervention such as thoracentesis); no Grade 4-5 complications or deaths within 90 days postoperatively |
| W. Yang2022 | 25% (5/20) | Anastomotic leakage/2 cases/10.0%; Pulmonary infection/1 case/5.0%; Postoperative hemorrhage/1 case/5.0%; Postoperative hoarseness (recurrent laryngeal nerve palsy)/1 case/5.0% | None |
| Z. Zhang2022 | Approximately 13.3% (6/45) | Pulmonary infection/3 cases/6.7%; Anastomotic leakage/1 case/2.2%; Incisional hernia/1 case/2.2%; Early postoperative death (hypovolemic shock)/1 case/2.2% | Present (2 cases, 4.4%); Grade 3 immune-related encephalitis (1 case, requiring glucocorticoid anti-inflammatory and antiepileptic drug treatment), Grade 5 early postoperative death from hypovolemic shock (1 case) |
| X. Chen2023 | 10% (3/30) | Recurrent laryngeal nerve injury/1 case/3.3%; Esophageal stricture/1 case/3.3%; Pneumothorax/1 case/3.3% | None; All complications were Grade 1; no 30-day mortality |
| J. He2023 | 15% (3/20) | Anastomotic leakage/2 cases/10.0%; Recurrent laryngeal nerve injury/1 case/5.0% | None |
| Y. Li2023 | Approximately 6.9% (4/58) | Experimental group: Anastomotic leakage + biliary obstruction/1 case/3.4%; Pneumonia/1 case/3.4% (no individual complications listed for control group) | None in the experimental group |
| G. Yang2023 | Approximately 21.4% (9/42) | Anastomotic leakage/8 cases/19.0%; Chylothorax/1 case/2.4% | None |
| Z. Zhang2021 | No clear total incidence rate (only individual statistics) | Pneumonia/15 cases/65%; Pleural effusion/10 cases/43%; Cardiac events (e.g., arrhythmia)/5 cases/22%; Anastomotic leakage (including 1 post-discharge readmission)/3 cases/13%; Chylothorax, recurrent laryngeal nerve palsy, postoperative hemorrhage/1 case each/4% | Present; Grade 3 pneumonia (9 cases, 39%, some requiring ICU monitoring), Grade 3 pleural effusion (2 cases, 9%, requiring thoracentesis); no Grade 4-5 complications or deaths within 30 days postoperatively |
| X. Yan2022 | 77.8% (28/36) | Anemia/16 cases/44.4%; Pneumonia/14 cases/38.9%; Pleural effusion/4 cases/11.1%; Arrhythmia, anastomotic leakage, wound infection/2 cases each/5.6%; Recurrent laryngeal nerve palsy/3 cases/8.3%; Chylothorax, anastomotic stricture, hypoalbuminemia, heart failure/1 case each/2.8% | Present (including 1 Grade 5 fatal event, incidence rate 2.8%); Several Grade 3 adverse reactions (pneumonia, pleural effusion, etc., requiring invasive intervention), Grade 5 death from anastomotic leakage with hemorrhage (1 case) |
| W. Xing2021 | Approximately 63% (15/24) | Thirteen patients developed postoperative pulmonary infection, 3 of whom were complicated with sepsis and 1 of whom died, while 2 patients developed anastomotic fistula. | There were 3 cases of Grade 3 or higher surgery-related adverse events, all associated with postoperative pulmonary infection, specifically including 2 cases of sepsis (Grade 3) and 1 case of death (Grade 5). |
| N. Zhou2024 | 28% (7/25) | Individual types not listed | None; All complications were mild Grade 1-2 events; no deaths within 90 days postoperatively; Grade 3 immune-related myocarditis during postoperative adjuvant therapy was unrelated to surgical procedures and not included |
| Y. Zheng2024 | 97.06% (99/102) | Pneumonia/76.47%; Pleural effusion/58.82%; Hoarseness/52.94%; Atelectasis/54.90%, etc. | Present (total incidence rate 20.59%); Grade III-IV: 18.63% (19 cases, including respiratory failure, anastomotic leakage, arrhythmia, etc., requiring medical intervention), Grade IIIb or higher: 9.8% (10 cases, severe complications requiring invasive procedures), Grade V (fatal): 1.96% (2 cases, deaths within 90 days postoperatively) |
| M. Wang2025 | Approximately 12.26% (13/106) | Pneumonia/6 cases/5.7%; Chylothorax/4 cases/3.8%; Wound infection/3 cases/2.8% | None; All complications were mild Grade 1-2; no fatal surgery-related adverse events within 30-90 days postoperatively |
| X. Sui2025 | Approximately 21.4% (3/14) | Pleural effusion/2 cases/14.3%; Empyema, esophagotracheal fistula, chylothorax/1 case each/7.1% (presence of combined complications) | Present (including 1 Grade 5 fatal event); Grade 5 death from esophagotracheal fistula (1 case, 7.1%); other complications were Grade 1-2 |
| H. Jiao2025 | 36.4% (20/55) | Main complications included infection, gastrointestinal reactions, etc. (individual incidence rates not subdivided) | Present (including 1 Grade 5 fatal event); Grade 5 perioperative death from severe infection (1 case, 1.8%); several Grade 3 or higher severe complications requiring medical intervention |
| J. Guo2025 | 38.1% (8/21) | Anastomotic leakage/4 cases/19.0%; Pleural effusion/3 cases/14.3%; Pneumonia/2 cases/9.5%; Recurrent laryngeal nerve palsy/2 cases/9.5% | No clear Grade 3 or higher severe events; 1 case of pneumonia progressed to sepsis but recovered after treatment; no Grade 5 fatal events |
| S. Deng2025 | 11.9% (5/42) | Anastomotic leakage/2 cases/4.76%; Pulmonary infection/3 cases/7.14% | None; All complications were mild to moderate Grade 1-2, cured with non-surgical intervention; no deaths within 90 days postoperatively |
| Y. Y. Chen2024 | Approximately 2.7% (1/37) | Severe postoperative pneumonia/1 case (no other clear records) | Present (1 Grade 5 fatal event); Grade 5 death from severe postoperative pneumonia (1 case, 2.7%); no other Grade 3-4 events |

**Supplementary Table 3.** Egger's tests for publication bias

| **Outcome Measure** | **t-value** | **Degrees of Freedom (df)** | **P-value** | **Bias Estimate (SE)** | **Tau²**  **(τ²)** |
| --- | --- | --- | --- | --- | --- |
| ORR | 1.69 | 12 | 11.7% | 1.818 (1.076) | 1.614 |
| pCR | -1.99 | 28 | 5.6% | -1.497 (0.752) | 1.604 |
| MPR | 1.86 | 18 | 8.0% | 2.218 (1.194) | 2.668 |
| TrAE | 5.31 | 11 | 0.0002* | 3.649 (0.687) | 1.154 |
| ≥ Grade 3 TrAE | -2.50 | 15 | 0.024* | -3.960 (1.583) | 4.701 |
| SrAEs | -2.72 | 19 | 0.014* | -2.8762(1.058) | 2.185 |
| ≥ Grade 3 SrAE | -3.12 | 19 | 0.006* | -1.9876(0.636) | 1.0823 |

**Note:** The publication offset of *R*_0_ was determined using the Beggs test, as the Egger test failed to converge. The results are as follows:

| **Test Name** | **Test Statistic (z)** | **P-value** | **Bias Estimate (SE)** |
| --- | --- | --- | --- |
| *R*_0_ | 0.18 | 0.86 | 8.0000 (45.3468) |
